# Rose Oil Distillation Wastewater: By-Products of Essential Oil Extraction as Circular Biostimulants for Tomato Growth

**DOI:** 10.3390/antiox14101252

**Published:** 2025-10-18

**Authors:** Nemanja Živanović, Ivana Danilov, Marija Lesjak, Tatjana Dujković, Nataša Simin, Vanja Vlajkov, Mirjana Ljubojević, Jovana Grahovac

**Affiliations:** 1 Faculty of Sciences, University of Novi Sad, 21000 Novi Sad, Serbia; nemanja.zivanovic@dh.uns.ac.rs (N.Ž.); marija.lesjak@dh.uns.ac.rs (M.L.); natasa.simin@dh.uns.ac.rs (N.S.); 2 Faculty of Technology Novi Sad, University of Novi Sad, Bulevar cara Lazara 1, 21000 Novi Sad, Serbia; tatjana.dujkovic@uns.ac.rs (T.D.); vanja.vlajkov@uns.ac.rs (V.V.); johana@uns.ac.rs (J.G.); 3 Faculty of Agriculture, University of Novi Sad, Trg Dositeja Obradovića 8, 21000 Novi Sad, Serbia

**Keywords:** circular economy, plant growth promotion, tomato, seed germination, seed treatment, antioxidant, gallic acid, quercetin, rutin, kaempferol

## Abstract

Rose processing into essentials oil is one of the major sectors providing inputs for cosmetics and health/food supplements industry, generating significant amount of wastewater if applying the steam distillation approach. Rose distillation wastewater (RDW), the major by-product of rose processing, still contains a significant load of polyphenolic compounds. This organic burden poses a significant environmental threat for RDW disposal, while, on the other hand, it still contains valuable compounds that could be valorized in the circular economy framework. This study has investigated the possibility of utilizing RDW in various concentrations (10%, 25%, 100% *v*/*v*) as a circular tomato growth biostimulant, addressing the existing research gap in the field of circular RDW valorization and its effects on plant growth modulation. LC-MS/MS and antioxidant assays have confirmed a rich antioxidant profile of RDW samples, with gallic acid, quinic acid, quercetin, kaempferol and their glycosides as the most abundant compounds. Tomato germination assays have resulted in significantly improved germination and initial seedling growth parameters when 10% RDW samples PA (‘Pure Aroma’), MA (‘Magic Aroma’) and NA (‘Natural Aroma) had been applied as seed treatment (10 seeds per treatment with each RDW), indicating varying plant growth-promoting potential depending on the RDW chemical composition. The increase in tomato growth parameters compared to the control varied in range 34% (MA)—60% (PA) for root length, 70% (MA)—109% (PA) for shoot length and 43% (MA)—72% (PA) for total seedling length, as well as 43% (MA)—72% (PA) for SVI-I and 40% (NA)—49% (MA) for SVI-II (seedling vigor indices I and II, respectively). Contrarily, the increase in RDW concentration of up to 25% and 100% (*v*/*v*) has resulted in inhibition of tomato germination and growth compared to the control (e.g., in range 10–50% (RDW 25%) and 45–87% (RDW 100%) for root length), suggesting the necessity for further optimization of RDW dosage in biostimulant applications. The results of this study open the field of possibilities for further development of circular plant biostimulants based on rose processing by-products, as value-added enrichment of the bio-based solutions portfolio for sustainable agriculture.

## 1. Introduction

The global demand for rose essential oil has seen significant growth in recent years, driven by increasing interest in natural and plant-based ingredients for cosmetics, food and wellness products. Valued at approximately USD 1.6 billion in 2022, the market is projected to reach USD 2.5 billion by 2032, with a compound annual growth rate of 4.56% from 2024 to 2032. Aromatherapy, wellness tourism and the preference for organic products continue to support this upward trend. Consequently, attention has shifted not only toward improving rose oil production technologies but also toward addressing sustainability and resource efficiency across the supply chain [1,2].

Rose essential oil is a premium product primarily consumed by major cosmetics and perfumery brands such as Chanel, Dior, Kenzo and others, with global annual consumption ranging from 3000 to 4500 kg. Approximately 90% of cultivated rose flowers are processed into essential oil, while 5–6% go into rose concrete and 3–4% into rose water. Bulgaria and Turkey dominate global production, supplying 80–90% of rose oil, largely due to favorable climatic conditions and long-standing cultivation traditions. Other contributors include Morocco, Iran, Mexico, France, Italy and Lebanon [3,4,5].

The most common method for extracting rose oil is industrial steam distillation, which uses large volumes of rose petals and generates substantial amounts of waste. For every kilogram of rose petals distilled, about 4 L of wastewater and 2 kg of wet petal residue are produced. A single distillation batch may use between 500 and 1000 kg of petals. Considering that it takes around 3000 kg of petals to produce just 1 kg of rose essential oil, global production results in an estimated 48 million liters of rose oil distillation wastewater (RDW) annually [6,7]. This by-product presents a significant environmental challenge and increases the overall cost of production.

Moreover, RDW is notably rich in polyphenolic compounds, naturally occurring substances that exhibit low biodegradability and can persist in the environment. If inadequately treated or improperly discharged, these compounds may function as bio-pollutants, potentially exerting harmful effects on aquatic ecosystems. Nonetheless, polyphenols are also well-documented for their antioxidant, antimicrobial and plant growth-promoting activities, as well as their ability to stimulate seed germination, making them valuable for applications in various industrial sectors [7,8,9,10,11,12]. Until now, there have been no established practices for the processing of RDW and utilization of present substances, such as polyphenols. This dual-nature environmental burden versus bioactive potential highlights the need for sustainable management and valorization of rose oil distillation by-products [4,5,7].

In this context, the present study investigates the potential application of RDW as a circular biostimulant for enhancing seed germination in tomato (*Solanum lycopersicum* L.). Despite its rich content of bioactive compounds, the use of this waste stream in seed germination remains largely unexplored. By repurposing this agro-industrial by-product as a value-added input in agriculture, the study aims to promote sustainable waste valorization within a circular economy framework. This approach supports environmentally friendly practices by reducing waste discharge and offering a green alternative for crop production enhancement [12].

Damask rose (*Rosa damascena* Mill.) is the primary species used for the commercial production of high-value rose essential oil due to its rich and distinctive aromatic profile. However, recent trends in breeding programs have shifted focus toward the aroma characteristics of garden roses (*Rosa × hybrida*), a widely cultivated ornamental species traditionally grown for decorative purposes. Unlike *R. damascena*, *R. × hybrida* has a broader geographic distribution and greater adaptability to different growing conditions [13]. Given this emerging interest and the species’ wider availability, *Rosa × hybrida* was selected as the raw material for this study to align with current research and industry directions.

## 2. Materials and Methods

### 2.1. Preparation of Rose Distillation Wastewater (RDW)

The rose distillation wastewater (RDW) was obtained as a by-product during the isolation of essential oil by the hydrodistillation method using a Clevenger apparatus in the laboratory setting. Rose petals of 8 garden rose genotypes (*Rosa* × *hybrida*) were collected in 2022 at the experimental fields of the Pheno Geno Roses Company (Temerin, Vojvodina, Serbia). Rose petals (about 170 g) were mixed with 1 L of tap water in a 2 L round-bottom flask and heated to boiling and maintained at that temperature for 3 h. After the collection of essential oils, the remaining RDW was left to cool, after which it was filtered. 100 mL of RDW was evaporated to dryness on the rotary vacuum evaporator (50 °C) and then reconstituted in 50% aq ACN (acetonitrile) to a concentration of 100 mg/mL and used for chemical characterization and antioxidant activity evaluation assays. The remaining RDW were stored in the freezer (−80 °C) until evaluation of their growth-promoting effects on tomato seeds.

### 2.2. Physico-Chemical Characterization of RDW

RDW samples and their corresponding dilutions used in seed germination assays (25% and 10%, *v*/*v*) were subjected to measurements of pH value and electrical conductivity (EC) using the multiparameter analyzer (C3010, Consort, Brussels, Belgium).

Total phenolic content (TPC) was determined by using the Folin–Ciocalteu reagent, according to the method described in the study by Simin et al. [9]. The analysis was conducted in triplicate, and the results were expressed as milligrams of gallic acid equivalents per gram of dry extract (mg GAE/g de) and as milligrams of gallic acid equivalents per liter of RDW (mg GAE/L).

Total flavonoid content (TFC) was determined by the colorimetric method with aluminum chloride, as described by Simin et al. [9]. The analysis was conducted in triplicate, and the results were expressed as milligrams of quercetin equivalents per gram of dry extract (mg QE/g de) and as milligrams of quercetin equivalents per liter of RDW (mg QE/L).

Total monomeric anthocyanin content (TAC) was determined by the pH-differential method as described in [9]. The analysis was conducted in triplicate, and the results were expressed as milligrams of cyanidin 3-*O*-glucoside equivalents per gram of dry extract (mg CE/g de) and as milligrams of cyanidin 3-*O*-glucoside equivalents per liter of RDW (mg CE/L).

**Quantitative analysis of selected compounds by LC-MS/MS.** The content of 45 selected compounds was investigated by liquid chromatography coupled with tandem mass spectrometry (LC-MS/MS), according to the method described in [14]. Samples and standards were analyzed using Agilent Technologies 1200 series high-performance liquid chromatograph, coupled with Agilent Technologies 6410A Triple Quad tandem mass spectrometer with electrospray ion source, and controlled by Agilent Technologies Mass Hunter Workstation software–Data Acquisition (ver. B.06.00) (Agilent Technologies, Santa Clara, CA, USA). RDW dry extracts dissolved in 50% aq ACN were diluted with 50% aq MeOH to the following concentrations: 20, 1, 0.1 and 0.01 mg/mL. A total of 5 µL of each dilution or standard was injected into the system, and compound separation was achieved using a rapid resolution column (Zorbax Eclipse XDB-C18, 50 mm × 4.6 mm, 1.8 µm—(Agilent Technologies, Santa Clara, CA, USA)). The column was thermostated at 50 °C, and the mobile phase, consisting of mobile phase solvents A (0.05% aq HCOOH) and B (MeOH) was delivered at a flow of 1 mL/min following the gradient: 0 min 30% B, 6 min 70% B, 9 min 100% B, 12 min 100% B, 3 min re-equilibration time (total run time is 15 min). Data were collected in a dynamic multiple reaction monitoring mode. Peak areas were determined using Agilent MassHunter Workstation Software–Qualitative Analysis (ver. B.06.00). Calibration curves were constructed by OriginLab Origin Pro (ver. 2019b) software and used for calculating the investigated compounds’ content. The LOD (limit of detection) and LOQ (limit of quantification) for all analyzed compounds are provided in the Supplementary Material (Table S1), as well as the standard curve equations along with R^2^ (coefficients of determination) for all quantified compounds (Table S2). The results were expressed as content: micrograms of compound per gram od dry extract (µg/g de) and as milligrams of compound per liter od RDW (mg/L).

### 2.3. In Vitro Antioxidant Activity of RDW

**DPPH scavenging assay.** Antioxidant activity of RDW was evaluated by their ability to scavenge 2,2-diphenyl-1-picrylhydrazyl (DPPH) radicals according to the method described by Simin et al. [9]. Analysis was conducted in triplicate, and IC_50_ values were determined (concentration of the sample that scavenges 50% of the radicals).

**The ferric reducing antioxidant potential (FRAP) assay** was used to further confirm RDW antioxidant activity. The analysis was performed following the method described in [9]. Analysis was conducted in triplicate, and results were expressed as milligrams of ascorbic acid equivalents per gram of dry extract (mg AAE/g de).

### 2.4. In Vitro Tomato Germination Tests

The tomato germination tests have been performed in 2025 at the Laboratory for Biochemical Engineering, Faculty of Technology Novi Sad, University of Novi Sad, Novi Sad, Serbia. The surfaces of tomato seeds (*Solanum lycopersicum* L., Maraton F1, seed lot number: 2–24/24, Superior, Velika Plana, Serbia) were sterilized using the sodium hypochlorite treatment, as previously described [15] and 10 seeds were placed in a Petri dish (90 mm) containing the filter paper, following the similar pattern of seed positioning in each Petri dish. The Maraton F1 variety was selected as one of the most abundant tomato varieties in Serbian market, which can yield 6–7 tomato fruits at each flowering branch, with an average fruit mass of 220 g. The sufficient level of moisture for seed germination was provided by adding 2 mL of sterile distilled water to filter paper in each Petri dish. Each Petri dish received a seed treatment of 1 mL containing RDW samples (non-diluted (100%), 4-fold (25%, *v*/*v*) and 10-fold (10%, *v*/*v*) diluted in sterile distilled water). RDW samples without any pretreatment performed have been used for seed treatment experiments. Negative controls included application of sterile distilled water in the same volume as the treatment. Each treatment was performed in a triplicate test. The Petri dishes were sealed with parafilm to prevent the moisture loss and incubated in the dark during the first 48 h at 25 °C, while the next 5 days followed the protocol, including 16 h light period and 8 h dark period under the same temperature conditions. Each day, the germination percent (GP, %) for each treatment and control was calculated by enumeration of germinated and non-germinated seeds, where the germination criterion of minimum 1 mm radicle length was applied. These data have also been used for estimation of mean germination time (MGT, days—Equation (1), where n_i_ is the number of newly germinated seeds on day d_i_, while N_7_ is the total number of germinated seeds after 7 day-germination) and germination rate index (GRI, seeds/day—Equation (2)—starting the measurement from the day 2). The blinding of the measurer was assured by applying the encrypted samples’ tags, while the encryption code was available only to the analysts performing the experimental data analyses. The experimental measurements also included seedlings fresh mass (FM, g), dry mass (DM, g—after drying at 80 °C until reaching a constant mass), total length (TL, mm), root length (RL, mm) and shoot length (SL, mm) after the 7 day-germination to calculate seedling vigor indices I and II (SVI-I and SVI-II—Equations 3 and 4, respectively, using the TL in cm, and DM in mg).

(1) $$MGT=\frac{\sum \left(n_{i}\cdot d_{i}\right)}{N_{7}}$$

(2) $$GRI=\sum \left(\frac{n_{2}}{d_{2}}\right)+\ldots \left(\frac{n_{7}}{d_{7}}\right)$$

(3) $$SVI-I=\frac{GP\cdot TL}{10}$$

(4) SVI−II=GP·DM·1000

### 2.5. Statistical Analysis

The data of all spectrophotometric and LC-MS/MS results were analyzed by one-way ANOVA followed by the post hoc Tukey’s Honest Significant Difference (HSD) test for multiple comparisons of means in order to determine whether the data obtained for different rose genotypes differed significantly from each other (Real Statistics Resource Pack add-in for Excel 2013). Statistical significance was set at *p* ≤ 0.05. Correlation factors between chemical composition and in vitro antioxidant activities were calculated using regression analysis in Excel 2013 (Microsoft, Redmond, WA, USA). Principal component analysis and hierarchical clustering analysis of results for chemical characterization, antioxidant activity and tomato seed growth-promoting ability were carried out using Past ver. 4.03 software.

The Statistica 13.3 software (Dell Technologies, Round Rock, TX, USA) was applied to evaluate the statistical differences between the tomato seed treatments and controls using the One-Way ANOVA and Duncan’s multiple range post hoc test, taking into account 95% confidence intervals.

## 3. Results

### 3.1. Screening of Physico-Chemical and Antioxidant Properties of Rose Distillation Wastewater

The physico-chemical properties of rose distillation wastewater (RDW) samples and their corresponding dilutions used in seed germination assays (25% and 10%, *v*/*v*) are presented in Table 1, including the results of pH value and electrical conductivity (EC) measurements. Only slight changes in pH values of RDW samples could be observed after the RDW dilution, while more prominent decreases in EC values could be observed after the RDW dilution.

The chemical composition of rose distillation wastewater (RDW) was evaluated by determining total phenolic content (TPC), total flavonoid content (TFC) and total monomeric anthocyanin content (TAC) (Table 2).

Investigated RDWs were very rich in phenolics with TPC in the range 78–193 mg GAE/g de, that is, 1195–3035 mg GAE/L RDW. RDW obtained from PA is characterized by the highest content of TPC, followed by MA, AA and MIF, while the lowest contents were recorded in RDWs from IA and UA. Moreover, RDWs were also rich in flavonoids with TFC in the range of 10.73–26.64 mg QE/g de, that is, 145.9–394.3 mg QE/L RDW. AA and PA RDWs were characterized by the highest content of TFC, followed by MIF and MA, while the lowest values were recorded for RDWs from NA, IA, UA and GA. Even though PA, MA, AA and MIF are pink colored roses, and PA, AA, and MIF are intensely colored, TAC could be determined only for PA and MIF RDWs. This suggests that high temperatures and long duration of isolation of the essential oil are conditions under which anthocyanins are not particularly stable and degrade. PA was characterized by a TAC of 0.68 mg CE/g de, that is, 10.74 mg CE/L RDW, while MIF showed a lower content of 0.23 mg CE/g de, that is, 3.75 mg CE/L RDW.

To further investigate the chemical content of RDWs, the content of 45 selected compounds was examined by LC-MS/MS, and 22 compounds were quantified; the results are shown in Table 3. All RDW samples were very rich in gallic acid with content in the range of 43.6–157 mg/g de, that is, 650–2564 mg/L RDW. MIF and MA were characterized by the highest content, while IA had the lowest content of gallic acid. Another compound was quinic acid, with a content ranging from 17.2 to 81.8 mg/g de, that is, 241–1030 mg/L RDW. The lowest amount was detected in MA, while the highest content was detected in GA. Protocatechuic acid was present in significant amounts in PA and AA, with content of 3.4 and 2.6 mg/g de, respectively, that is, 53.0 and 39.1 mg/L RDW, respectively. Other phenolic acids were present in trace amounts.

When it comes to flavonoids, flavonols quercetin and kaempferol and their glycosides were present in high amounts. The content of kaempferol was in the range from 0.06 to 1.1 mg/g de, that is, 0.8–16.23 mg/L RDW. The highest amount was detected in IA and GA, while the lowest content was recorded for MA. The content of kaempferol 3-*O*-glucoside was in the range 3.3–44.3 mg/g de, that is, 51.3–660 mg/L RDW. The highest content was recorded for IA, while the lowest content was recorded for PA and MA. The content of kaempferol 3-*O*-glucoside showed a similar trend to the content of kaempferol. Like kaempferol, quercetin was present in significantly smaller amounts (0.06–1.6 mg/g de) compared to its glycosides quercitrin (0.2–16.9 mg/g de), hyperoside + isoquercetin (0.6–16.6 mg/g de) and rutin (0.04–0.04–1.4 mg/g de). It is interesting to note that IA, UA and GA, which had the highest contents of kaempferol and kaempferol 3-*O*-glucoside, also had the lowest amounts of quercetin and its glycosides. PA and MA were characterized by high contents of quercetin glycosides and low amounts of kaempferol 3-*O*-glucoside, while AA and MIF had high contents of all flavonoid glycosides.

Analyzed compounds that were not detected or below LOQ (limit of quantification) in all samples were cinnamic acid, 3,4-dimethoxycinnamic acid, daidzein, genistein, epicatechin, myricetin, matairesinol, secoisolariciresinol, gentisic acid, o-coumaric acid, umbelliferon, esculetin, scopoletin, syringic acid, apigenin, luteolin, isorhamnetin, apigenin 7-*O*-glucoside, vitexin, baicalin, luteolin 7-*O*-glucoside, epigallocatechin gallate and apiin. LOD (limit of detection) and LOQ of the LC-MS/MS method for quantitative determination of all selected compounds are provided in Supplementary Materials—Table S1. Standard curve equations and R^2^ (coefficients of determination) for all quantified compounds in RDW samples by LC-MS/MS are provided in Supplementary Materials—Table S2.

The results for antioxidant properties of RDW, determined by the DPPH and FRAP assays, are presented in Table 4. All RDWs showed strong antioxidant activity with IC_50_ values for the DPPH assay ranging from 7.1 to 19.4 μg de/mL. The highest antioxidant activity was shown by AA, followed by MIF, PA and NA, while the weakest antioxidant activity was shown by IA. In the FRAP assay, values were in the range 83.3–183.2 mg AAE/g de. Strongest activity exhibited MIF and PA, followed by MA and AA, while the weakest antioxidant activity was recorded for IA. There was a strong correlation between TPC, TFC, and gallic acid content and activity in DPPH (with regression factors R^2^ 0.951, 0.944, 0.945, respectively) and FRAP assays (with regression factors R^2^ 0.976, 0.934, 0.923, respectively). Lower levels of correlation were between content of quinic acid, hyperoside + isoquercetin, quercitrin, rutin, naringenin and p-coumaric acid and DPPH (with regression factors R^2^ 0.730, 0.788, 0.738, 0.626, 0.787 and 0.728, respectively) and FRAP (with regression factors R^2^ 0.742, 0.739, 0.717, 0.719, 0.642, 0.649, respectively) antioxidant activity. Based on these correlation factors, it can be said that antioxidant activity is greatly dependent on the content of gallic acid, the most abundant phenolic compound in samples. To further confirm the gallic acid contribution to antioxidant activity, partial correlation factors were calculated with FRAP and DPPH, while controlling for TPC, and the obtained partial correlation factors were 0.56912 and 0.72728, respectively. Based on these correlation factors, it can be concluded that gallic acid acts in synergy with other phenolic compounds regarding the antioxidant activity.

### 3.2. Tomato Growth Parameters After Seed Treatment Using Rose Distillation Wastewater Samples

**Figure 1:**
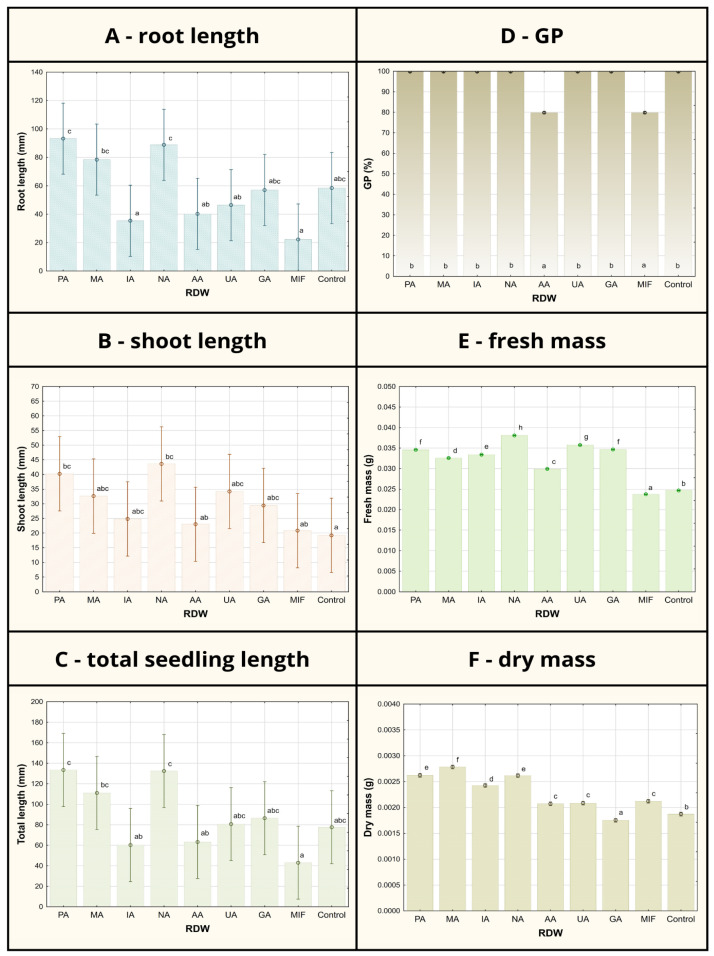
Tomato germination/growth parameters after 7-day seed treatment using various samples of rose distillation wastewater (RDW): PA—‘Pure Aroma’, MA—‘Magic Aroma’, IA—‘Intense Aroma’, NA—‘Natural Aroma’, AA—‘Adore Aroma’, UA—‘Unique Aroma’, GA—‘Gentle Aroma’, MIF—‘Mina Frayla’. GP designates germination percent. Letter designations represent different levels of statistical significance based on Duncan’s multiple range test. Values marked with the same letter are at the same level of significance (95% confidence interval).

**Figure 2:**
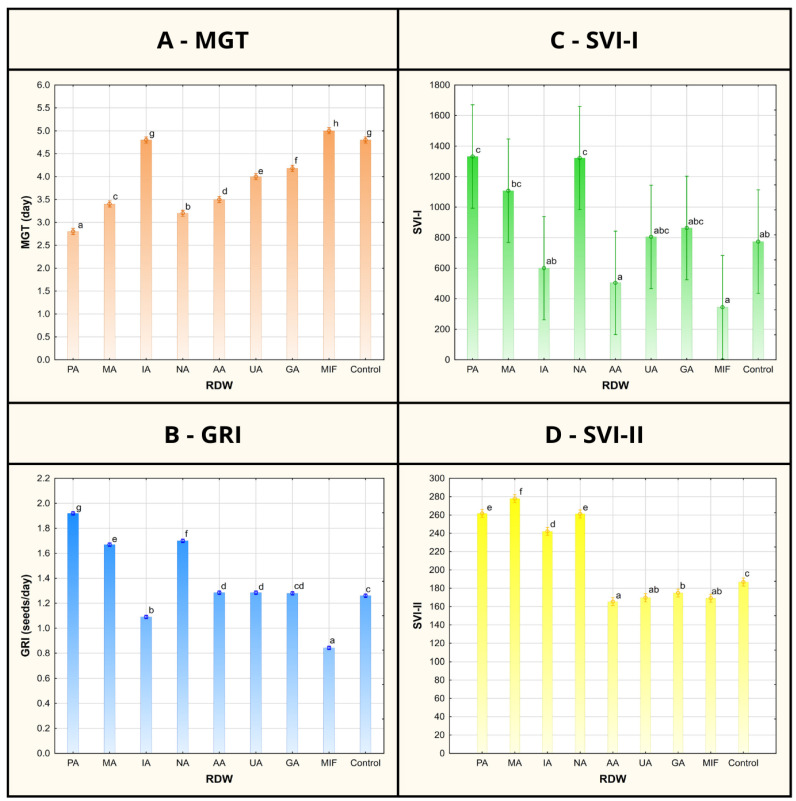
Mean germination time (MGT), germination rate index (GRI) and seedling vigor indices I and II (SVI-I and SVI-II) of tomato seeds/seedlings after treatment using various samples of rose distillation wastewater (RDW): PA—‘Pure Aroma’, MA—‘Magic Aroma’, IA—‘Intense Aroma’, NA—‘Natural Aroma’, AA—‘Adore Aroma’, UA—‘Unique Aroma’, GA—‘Gentle Aroma’, MIF—‘Mina Frayla’. Letter designations represent different levels of statistical significance based on Duncan’s multiple range test. Values marked with the same letter are at the same level of significance (95% confidence interval).

Investigation of PGP traits of RDW on the growth of tomato plants, taking into account seed germination phase and the initial seedling growth phase, was carried out using different concentrations of RDW samples (10%, 25% and 100%, *v*/*v*). Considering that the best results were achieved using RDW concentration of 10% in each individual case when it comes to tomato growth promotion, these results were presented in the study as follows (Figure 1 and Figure 2), while the rest of the results related to tomato growth parameters measured after treatment with RDW’s concentrations of 25% and 100% were provided in the Supplementary Material (Table S3).

Considering the root length, shoot length and total seedling length, the most prominent tomato growth-promoting effects were observed in case of RDW samples PA, MA and NA, in which application resulted in significantly increased seedling growth parameters compared to the control (Figure 1). Sample PA has contributed to the improvement in root length of 60%, shoot length of 110% and the overall seedling length of 72% compared to control. Similar results were obtained in case of sample NA: 52% increase in root length, 171% increase in shoot length and 71% increase in the total seedling length compared to the control. Improvement in the tomato growth parameters observed in case of sample MA used as seed treatment has been recorded as follows in comparison to the control: 34% for root length, 67% for shoot length and 43% for the total seedling length, exhibiting slightly lower potential for tomato growth promotion in comparison to samples PA and NA. On the other hand, some of the tested samples (UA and GA) have resulted in tomato growth parameters comparable to the control (grouped at the same level of statistical significance based on Duncan’s multiple range test in case of root length and total seedling length), while the samples IA, AA and MIF have inhibited tomato growth, resulting in the lower mean total seedling length compared to the control, arising mostly from inhibition of root development.

The record of germination percentage (GP) of tomato seed has shown uniformity between the control and majority of the RDW-based treatments (at 100%), except for the treatments based on samples AA and MIF, resulting in inhibition of tomato seed germination (Figure 1). Mean germination time (MGT), representing the average time required for seeds to start germinating, was found to be the shortest in case of samples PA (2.8 days), NA (3.2 days) and MA (3.4 days) and significantly shorter in comparison to the control (4.8 days) (Figure 2). Treatments including samples AA, UA and GA have also resulted in shorter MGT compared to the control, while the treatments IA and MIF have resulted in similar or longer MGT compared to the control. The similar results were observed in case of germination rate index (GRI), reflecting the seed vigor based on speed and completeness of seed germination under a certain treatment, where the best results were achieved in case of the treatments based on samples PA (1.92 seeds/day), NA (1.70 seeds/day) and MA (1.67 seeds/day) in comparison to the control (1.26 seeds/day), while other treatments achieved similar (AA, UA and GA) or lower values of GRI (IA and MIF) compared to the control.

Less differences between the treatments could be observed in the case of fresh and dry mass of tomato seedlings when compared to previously discussed tomato growth parameters (Figure 1). All treatments except the treatment based on sample MIF have resulted in improved fresh mass of tomato seedlings compared to the control, with the most prominent improvements observable in case of samples NA (54%), UA (45%) and GA (40%). On the other hand, only the treatment based on sample GA has resulted in a lower mean value of tomato seedlings dry mass compared to the control, while the most significant improvements were recorded for treatments MA (47%), PA (37%) and NA (37%).

While GP, MGT and GRI focus on parameters indicating seed vigor, newly formed seedling vigor is reflected through seedling vigor indices I and II (SVI-I and SVI-II), taking into account both GP and average length of seedlings (TL) or their average dry mass (DM). The highest values of SVI-I were achieved in the case of treatments based on samples PA (1334), NA (1324) and MA (1110), presenting 72%, 71% and 43% improvement compared to the value of SVI-I obtained for the control (776), respectively (Figure 2). Higher values of the SVI-I compared to the control were also recorded in case of the treatments UA and GA, while the treatments IA, AA and MIF resulted in lower tomato seedling vigor based on SVI-I compared to the control seedlings. A similar situation could be observed when shedding light on SVI-II, where the highest values of the target index were observed for treatments MA (278.40), PA (262.48) and NA (261.68), providing significant improvements of 49%, 40% and 40%, respectively, compared to the control. Besides the aforementioned treatments, only the treatment based on sample IA scored a higher value of SVI-II than the control.

**Figure 3:**
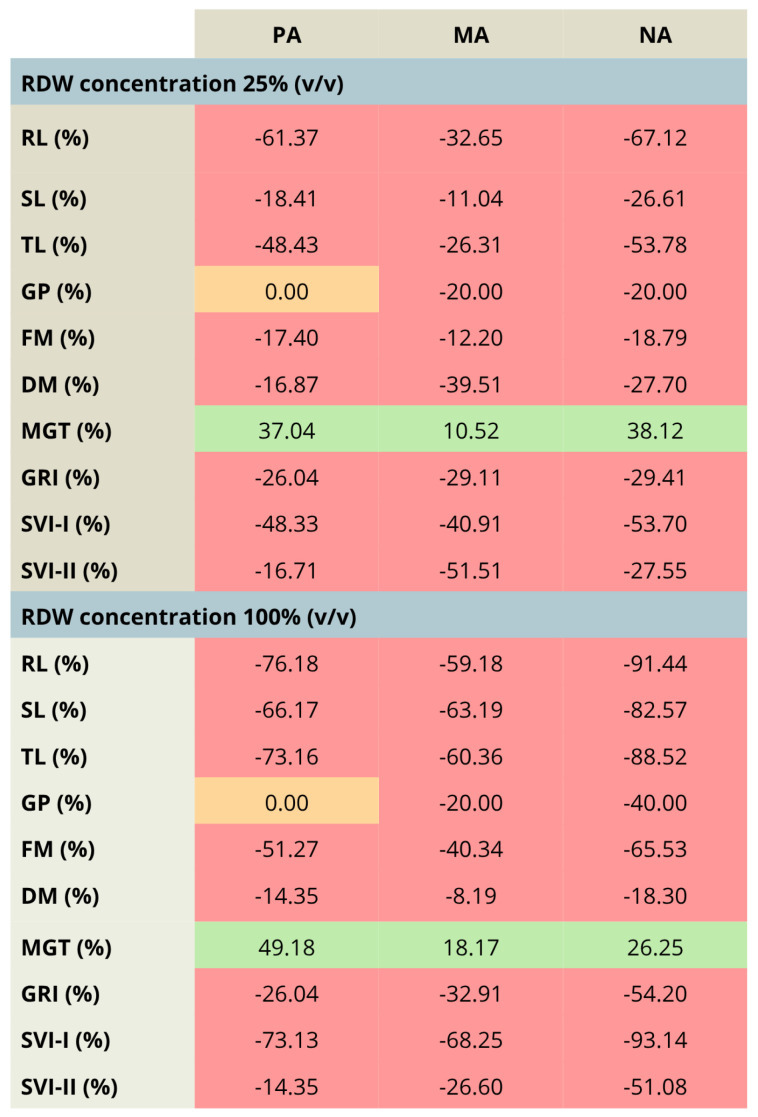
Percentage changes in the values of tomato growth/germination parameters—comparison between the increased rose distillation wastewater concentration (RDW—25% and 100% (*v*/*v*)) and the RDW concentration 10% (*v*/*v*), used as a baseline, presented for RDW samples PA—‘Pure Aroma’, MA—‘Magic Aroma’ and NA—‘Natural Aroma’. Red fields—decreased values of the tomato growth/germination parameters, orange fields—unchanged values of the tomato growth/germination parameters, green fields—increased values of the tomato growth/germination parameters. RL—root length, SL—shoot length, TL—total seedling length, FM—fresh mass, DM—dry mass, GP—germination percent, MGT—mean germination time, GRI—germination rate index, SVI-I and SVI-II—seedling vigor indices I and II.

The comparison between the treatments involving the aforementioned concentration range of RDW (10–25–100%) has been highlighted in Figure 3, presenting the percentual changes in tomato growth parameters for the most prominent RDW samples PA, MA and NA, taking into account the treatment with 10% RDW as a baseline. The results presented in Figure 3 clearly present that the increase in RDW concentration has resulted in tomato growth inhibition, as could be observed by the decrease in each growth parameter, except mean germination time (MGT), whose increase indicates slowed down germination. The only exception was observed in the case of germination percent for the treatment PA, which remained unchanged independent of the applied RDW concentration.

### 3.3. Principal Component Analysis

**Figure 4:**
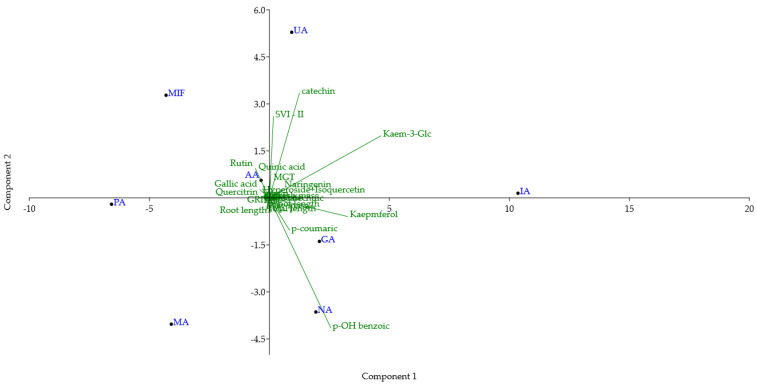
Biplot of principal component analysis (PC1 vs. PC2) of TPC, TFC, 14 identified compounds, FRAP, DPPH antioxidant activity and 10 parameters of tomato growth for rose distillation wastewater samples. PA—‘Pure Aroma’, MA—‘Magic Aroma’, IA—‘Intense Aroma’, NA—‘Natural Aroma’, AA—‘Adore Aroma’, UA—‘Unique Aroma’, GA—‘Gentle Aroma’, MIF—‘Mina Frayla’.

Principal component analysis (PCA) was conducted on a dataset comprising TPC, TFC, and the content of 14 compounds quantified in all RDW samples, DPPH and FRAP assay antioxidant activities, and 10 parameters examined for the tomato seed growth-promoting effect of RDW (Figure 4). The first and second principal components (PC1 and PC2) accounted for 57.47% and 20.78% of the total variance, respectively. This indicates significant differences in the chemical composition and biological activities among the examined RDW samples. Samples PA and MA are positioned in the lower left quadrant, due to their low content of catechin, kaempferol and kaempferol 3-*O*-glucoside and high contents of rutin and quercitrin. MIF is positioned in the upper left quadrant due to high content of rutin, catechin, lower amounts of kaempferol and kaempferol 3-*O*-glucoside, and low amounts of *p*-hydroxybenzoic and *p*-coumaric acids. Sample AA is positioned in the upper left quadrant, close to the center of the biplot. Compared to MIF, it is characterized by a higher content of quercitrin, kaempferol, kaempferol 3-*O*-glucoside, *p*-hydroxybenzoic and *p*-coumaric acids, and a lower amount of catechin. UA is positioned in the upper right quadrant close to the border with the left one. It is characterized by the high content of catechin and kaempferol 3-*O*-glucoside, higher content of rutin, and lower content of quercitrin. IA is positioned in the far-right part of the upper right quadrant, close to the border of the bottom one, due to the highest amount of kaempferol 3-*O*-glucoside among the examined samples. Additionally, it has a high amount of kaempferol and a low amount of quercitrin and rutin. Samples GA and NA are positioned in the lower right quadrant due to the higher content of kaempferol and kaempferol 3-*O*-glucoside, with low levels of catechin. NA is positioned in the lower part of the quadrant due to the lower content of rutin, kaempferol, and higher content of *p*-hydroxybenzoic acid.

**Figure 5:**
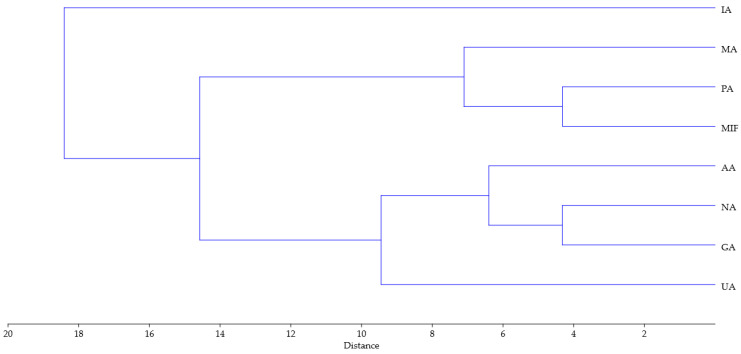
Dendrogram obtained using hierarchical clustering analysis of TPC, TFC, 14 compounds, FRAP, DPPH antioxidant activity and 10 parameters of tomato growth for rose distillation wastewater samples. PA—‘Pure Aroma’, MA—‘Magic Aroma’, IA—‘Intense Aroma’, NA—‘Natural Aroma’, AA—‘Adore Aroma’, UA—‘Unique Aroma’, GA—‘Gentle Aroma’, MIF—‘Mina Frayla’.

Similar conclusions can be drawn from the dendrogram obtained using hierarchical clustering analysis of the same data (Ward’s method was used with Euclidean distance for measurement of closeness, Figure 5). Similarly to the PCA biplot, sample IA is separated from the other samples, while the rest can be divided into two groups, one comprising PA, MA and MIF, and the other of NA, GA, UA and AA.

## 4. Discussion

Rose extracts and essential oils are thoroughly studied in scientific literature and traditionally widely applied due to their health benefits, including anti-inflammatory and pain relief action, antioxidant, antimicrobial, antiviral and antitumor activity [16], contribution to digestive, cardiovascular and skin health, as well as mental and emotional well-being [17,18]. Rose distillation wastewater (RDW), the major rose processing by-product expected to contain a significant share of bioactive compounds (non-volatile or with low volatility), has been investigated for potential medicinal purposes [4,5,19]. On the other hand, its application for plant growth stimulation purposes has scarcely been investigated in scientific literature. To the best of the authors’ knowledge, this is the first study investigating the plant growth modulation effects of *Rosa × hybrida* RDW in tomato plants being investigated as seed treatment. The current state of the art reveals investigation of RDW as an organic amendment in combination with goat manure, compost, mycorrhizae and plant growth-promoting (PGP) rhizobacteria in the organic greenhouse cultivation of Damask rose, where a significant improvement in rose growth parameters has been recorded [20]. So far, RDW has often been valorized via composting or use in animal forage [21], with novel possible valorization routes emerging through advanced strategies for recovery of bioactive antioxidant compounds [6,22], their incorporation in bioplastic-based edible films for food packaging [10] or microbial bioconversion to natural bioactive aroma compounds [23].

### 4.1. Chemical Profiling and Antioxidant Potential of RDW Obtained from Various Rosa Cultivars

RDWs examined in this study were rich in phenolic compounds, which is no surprise when they are compared to the content of TPC in methanol petal extracts from roses grown in Vojvodina (91.4–217 mg GAE/d de and 148–260 mg GAE/g de) and TFC (17.3–56.3 and 23.7–26.8 QE/g de). Differences in content are the result of differing genotypes, year of flower collection and extraction method [9,24]. RDWs can be a rich source of phenolics, which was shown in the paper by Sabahi et al. [22], where the examined wastewater had TPC of 167.7 mg GAE/g de, TFC of 29.9 mg QE/g de and TAC of 0.31 mg/g de [22], with TPC and TFC levels very similar to the PA, AA and MIF from this study. RDW from Andre Rieu rose was characterized by a high TPC of 153 mg GAE/g de, low TFC of 1.92 mg QE/g de and significantly higher TAC (1.92 mg CE/g de), compared to RDWs in the present study [10]. Dudonné and collaborators examined the hot water extract of *R. damascena* and recorded TPC of 124.9 mg GAE/g de, similar to RDW of GA from this study [25]. The TAC of PA, AA and MIF (0.68; <0.40 and 0.23 mg CE/g de, respectively) is significantly lower compared to methanol petal extracts of the same roses (9.19, 13.1 and 2.21 mg CE/g de), suggesting that anthocyanins are highly degraded under the conditions of hydrodistillation of essential oil [24,26].

Phenolic profile determined by LC-MS/MS revealed significant differences compared to methanol extracts. PA, AA and MIF rose methanol extracts are characterized by gallic acid content of 22.6, 29.3 and 30.4 µg/g de, respectively [24]. On the contrary, RDWs of PA, AA and MIF had content of 76.4, 149.9 and 157.3 mg/g de. This difference could be a result of the extraction at high temperature. RDWs from this study are rich in flavonoids kaempferol and quercetin and their glycosides, similarly to methanol extracts of rose petals from roses grown in Vojvodina, Serbia [9,24]. Compared to Andre Rieu RDW, samples from this study show higher content of kaempferol and quercetin glycosides, further showcasing metabolic differences between these genotypes [10]. A similar trend was reported by Rusanov and collaborators. In their study, RDW from *R. damascena* was rich in quercetin and kaempferol glycosides [6]. Other studies also reported quercetin and kaempferol as dominant flavonoids in rose petals [27,28,29].

The RDWs showed strong antioxidant activity, which is to be expected, because of the high content of phenolic compounds such as gallic acid and flavonoids, which are known for antioxidant activity. RWD had a similar ability to neutralize DPPH radicals (IC_50_ in the range 7.1–19.4 µg/mL) compared to methanol extracts of rose petals from roses grown in Serbia (IC_50_ in the range 9.24–42.5 µg/mL and 17.7–27.8 µg/mL). It can be noted that RDWs of PA, AA and MIF have lower IC_50_ values compared to methanol extracts of petals from the same cultivars [9,24]. A similar trend can be seen for the Andre Rieu cultivar with IC_50_ of 22.7 and 2.85 µg/mL for the methanol extract and RDW, respectively [10]. Rose extracts exhibited weaker DPPH radical scavenging activity than RDWs from the present study, with IC_50_ values in the range 0.08–5.78 mg/mL [30]. Rose essential oil distillation wastewater in the study by Sabahi et al. had an IC_50_ of 226.7 µg/mL. In the same study, quercetin showed strong antioxidant activity with an IC_50_ value of 26.5 µg/mL. RDWs from the present study are not just numerous times better antioxidants than wastewater but are also stronger antioxidants than quercetin [22]. In the FRAP assay, RDWs also showed strong antioxidant activity with values in the range 83.3–183.2 mg AAE/g de. In our previous studies, rose petals’ methanol extracts had activities in the range 74.6–227 and 132–209 mg AAE/g de [9,24]. Based on the results from both assays and the literature data, RDWs from the present study are excellent antioxidants.

### 4.2. Plant Growth Modulating Capability of RDW

In the present study, RDW samples obtained from different rose cultivars were investigated as the potential biostimulants for tomato growth in the initial growth phases via seed treatment, using a range of different RDW concentrations (10–25–100%, *v*/*v*). Based on the results presented, an obvious interaction between RDW concentrations and various plant growth parameters could be established, leading to the conclusion that the lowest tested concentration of RDW (10%) resulted in the most prominent tomato growth promotion. In other words, various inhibitory effects to tomato growth could be observed when applying 25% of RDW or pure RDW, ranging from inhibition of root and shoot development to decreased germination percentage. Similar results were observed when investigating tomato growth modulatory effects of plant extracts, such as extracts of *Juniperus sabina*, *Allium jesdianum*, *Dorema aucheri*, *Taraxacum officinale*, *Rheum ribes* and *Conocarpus erectus*, where the decrease in various growth parameters has been recorded with the increasing concentration of plant extracts [31]. Considering the only slight increase in pH values of the diluted RDW samples presented in Table 1, low pH values of the undiluted RDW samples could not be classified as the factor contributing to tomato growth inhibition. However, considering the strong buffering capacity and acidic nature of RDW, further research should be conducted to investigate plant growth-modulating effects of RDW with pH values modified toward the neutral range. On the other hand, varying tomato growth modulatory effects were also observed among different RDW samples applied as seed treatment in similar concentration (10%). The most prominent increase in tomato growth parameters compared to the control was observed with the treatments with PA, NA and MA, while application of other samples either resulted in growth parameter values comparable to the control or tomato growth inhibition. Hence, a closer look into the chemical composition of the investigated RDW samples has been made to better understand the possible chemical triggers of the various tomato growth responses recorded across the span of the investigated RDWs. First, the general mechanisms of plant growth modulation by plant biostimulants were discussed, with a later deeper dive into antioxidant chemical profiling of the specific RDWs promoting tomato growth.

### 4.3. A Roadmap for Further Research and Validation Steps: The Proposed Mechanisms of Plant Growth Promotion in Relation to RDW Chemical and Antioxidant Composition

Although a significant research gap is present in the field of RDW application as a plant biostimulant, some general mechanisms of plant growth modulation based on the activity of specific antioxidant compounds present in both RDW and other plant extracts could be considered as possibly responsible for tomato growth modulation in this study.

The application of botanical extracts can influence plant growth through both direct and indirect pathways. A direct mechanism involves the regulation of internal plant processes, such as nutrient acquisition and hormonal signaling. Plant extracts can enhance nutrient acquisition and use efficiency, which is a key characteristic of biostimulants, by providing directly usable plant nutrients. The most important nutrients contained in rose tissues comprise vitamins C and B [32], whose exogenous application can enhance plant growth and stress tolerance [33], as well as minerals, which could serve as micronutrient sources in trace concentrations present in RDW [34]. Roses naturally produce various plant hormones, including auxins, cytokinins, gibberellins, abscisic acid, ethylene, etc. [35], and it is plausible that RDW could contain trace amounts of these [36]. These hormones are vital and directly influence cell division, elongation, root development, and overall plant growth. An additional, indirect mechanism involves the interaction of RDW with the plant and soil microbiome. This suggests that RDW would not only provide bioactive compounds for direct plant absorption but also foster a more favorable microbial community around the plant roots or at the plant surface, opening the pool of possibilities for indirect microbially mediated PGP. Solid remains of rose tissues present in RDW could serve this purpose, considering their insolubility in water, presenting the possible inducers of soil microbial enzymatic activity for degradation of complex substrates.

When plants are subjected to stress, they experience oxidative damage. To counteract this, they naturally increase the activity of antioxidant enzymes like catalase, peroxidase and superoxide dismutase [37]. The exogenous application of RDW, rich in its own potent antioxidants, could supplement the plant’s internal defense system. This would allow the plant to more effectively protect its cells from oxidative damage, freeing up metabolic resources that would otherwise be dedicated to stress response. This redirection of energy could then be utilized for growth and development, leading to a healthier, more productive plant.

A crucial principle in the study of botanical biostimulants is the ‘synergy hypothesis’. Research indicates that the beneficial effects of plant-based biostimulants are often not due to a single active substance, but to the coordinated action of multiple compounds [38]. In many cases, crude plant biostimulants exhibit greater overall bioactivity compared to their individual constituents when applied individually. RDW is by its very nature a complex, multi-compound mixture. The collective and synergistic action of its diverse phytochemicals could produce a more powerful and holistic PGP effect than any single, isolated compound. This principle is what elevates the potential of RDW beyond the mere sum of its parts. For example, the combined effect of multiple antioxidant compounds might offer a broader and more robust defense against a variety of stresses than a high concentration of a single antioxidant [39]. This perspective frames the lack of direct research on the ‘whole RDW’ as a significant scientific void, as a study on any single isolated compound would fail to capture the full scope of its potential efficacy. The compelling proposition, therefore, was to investigate the RDW as a complete, complex biostimulant system rather than a simple solution of isolated chemicals, still maintaining a focus on the previously known possible PGP mechanisms related to and proven to be induced by the individual bioactive compounds present in RDW.

Rose tissues, especially rose petals, are a storehouse of diverse phytochemicals, notably phenols, flavonoids, anthocyanins and carotenoids, with concentrations varying by cultivar and rose cultivation conditions [17]. There is a strong scientific consensus that plant extracts function as biostimulants primarily by enhancing a plant’s resistance to environmental challenges like drought, salinity, and temperature fluctuations, a process heavily mediated by antioxidant compounds [40]. The state-of-the-art research outcomes highlight a strong link between the antioxidant capacity of plant extracts used as biostimulants and plant’s ability to confer stress tolerance [36]. Considering that RDW could be classified as a rose extract, its strong antioxidant profile, which is directly and strongly correlated with their content of anthocyanins, flavonoids and polyphenols [8,22,27], could be investigated for plant growth biostimulating effects. Taking a closer look at the chemical profile of RDW analyzed in this study, a higher abundance of several bioactive compounds could be observed across the range of the tested RDW samples, including gallic acid, quinic acid, protocatechuic acid, kaempferol, kaempferol 3-*O*-glucoside, quercitrin, quercetin, hyperoside + isoquercetin and rutin.

Gallic acid was previously investigated for its plant growth-promotion traits, deciphering a significant dose dependence of the plant growth parameters, primarily influencing plant growth via auxin signaling, antioxidant defense, and stress alleviation [41]. The previously reported content of gallic acid in wastewater of Narcea rose hydrodistillations was 726.6 µg/g [8], with a significantly lower value compared to this study, where the gallic acid content in various RDW samples ranged from 650.1 ± 58.51 mg/L RDW (43,632 ± 3927 µg/g de) in IA to 2564 ± 230.8 mg/L RDW (157,329 ± 14,160 µg/g de) in MIF. Several studies suggest that lower concentrations of gallic acid can slightly stimulate primary root growth; conversely, excessive concentrations of gallic acid profoundly reduce primary root length and root meristem size, likely by suppressing cell division, mostly due to interference with auxin signaling, resulting in the decrease in auxin content in root tips by down-regulating the expression of auxin transporters [41]. Furthermore, it was reported that gallic acid, or derivatives synthesized from gallic acid as a precursor, such as ethyl crotonate ester of naphthophenone derivative, can exhibit auxin-like growth-promoter activity, particularly in relation to root development, suggesting that gallic acid might directly influence auxin pathways or act as an auxin-mimicking compound [42]. Inhibitory effects of RDW samples MIF and AA, with the highest content of gallic acid, on tomato growth were also observed in this study. On the contrary, the RDW sample MA, containing the comparable amount of gallic acid to MIF and AA, significantly positively influenced tomato growth, proving the necessity to further elucidate interactions among various identified bioactive compounds and their synergistic/antagonistic effects on tomato growth. Gallic acid is also a potent antioxidant, which, when applied to plant exogenously, has been shown to improve plant growth and photosynthetic activity under stress via various mechanisms: enhancing the antioxidant defense system by increasing the activity of antioxidant enzymes (e.g., catalase, peroxidase) and the accumulation of non-enzymatic antioxidants (e.g., ascorbate, glutathione, other phenolic compounds), improving osmotic and ionic homeostasis (e.g., by decreasing Na^+^ content and Na^+^/K^+^ ratio under salt stress) [43], promoting root growth and overall vigor in plants subjected to salt stress [44,45], as well as inducing plant defenses against pests and pathogens; for example, by activating jasmonic acid signaling [46]. Gallic acid and its derivatives have also been shown to improve tomato yield under biotic tomato stress caused by *Alternaria solani*, suggesting their antifungal and biocontrol potential [47].

Quinic acid is a key intermediate in the shikimate pathway, which is crucial for the biosynthesis of aromatic amino acids (phenylalanine, tyrosine, tryptophan) and a vast array of secondary metabolites, including phenolic compounds. Hence, its indirect PGP contribution could be attributed to the precursor role via synthesis of many compounds essential for plant growth and development, as well as those involved in defense mechanisms and stress responses [48]. It is also involved in metabolism of chlorogenic acids, known for their antioxidant potential and abiotic stress-responsiveness in plants [49]. While quinic acid is vital for plant metabolism internally, its reported direct plant growth-promoting effects when applied exogenously are less extensively documented. The reported content of quinic acid in RDW samples in this study was in range from 241.5 ± 24.15 mg/L RDW in MA to 1030 ± 103.0 mg/L RDW in GA, suggesting a possible dose-dependent inhibition of tomato growth, due to the recorded decrease in tomato growth parameters in RDW treatments with higher quinic acid content.

Protocatechuic acid was previously investigated as the potential agent for plant abiotic stress mitigation due to its interactions with the plant antioxidative response system. It contributed to the improvement in rye seedlings’ resistance to freeze–thaw cycles and the stress related to the presence of heavy metals (particularly Cd) by directly affecting the increase in plant antioxidant response enzymes (peroxidase, superoxide dismutase and catalase) and the content of soluble proteins, while simultaneously contributing to the reduction in malondialdehyde content [50]. Similarly, the improvement in antioxidative and photosynthetic processes of rice seedlings during their submergence resulted in improved shoot growth and survival percentage, as a result of the joint exogenous application of vanilic acid and protocatechuic acid in the concentration range 0.1–1 mM [51], which corresponds to the concentration range of protocatechuic acid in RDW samples in this study, except for the samples PA and AA.

Kaempferol is another prominent flavonoid, which is well-known as a potent antioxidant and signaling molecule. Similarly to quercetin and gallic acid, kaempferol can influence auxin transport and signaling pathways [52]. Flavonoids are known to interact with auxin efflux carriers (PIN proteins), thereby regulating auxin distribution and affecting root architecture and overall plant development [53]. This study identified varying content of kaempferol in RDW samples, ranging from 0.803 ± 0.056 mg/L RDW (57.35 ± 4.015 µg/g de) in MA to 16.23 ± 1.13 mg/L RDW (1089 ± 76.23 µg/g de) in IA, suggesting that lower content of kaempferol contributes to tomato growth promotion. Significantly lower kaempferol content was detected in wastewater obtained after Narcea rose hydrodistillation—13.76 µg/g [8]. Recent research has uncovered a fascinating role for kaempferol in the biosynthesis of ubiquinone (coenzyme Q), a vital respiratory cofactor found in both prokaryotic and eukaryotic respiratory chains and in photosynthesis [54]. It has been shown that the B-ring of kaempferol can be incorporated into ubiquinone through peroxidative cleavage [55]. This highlights how kaempferol, a specialized metabolite, can directly contribute to the synthesis of a primary metabolite essential for energy production and overall plant vitality [54]. Furthermore, its PGP effects might be enhanced in combination with other compounds, leveraging synergistic interactions. For instance, studies on potato have shown combined application of kaempferol and caffeic acid with plant growth-promoting rhizobacteria to be highly effective in mitigating salinity stress [56]. The aforementioned study applied kaempferol in the dosage 20 µM (5.7 mg/L), corresponding to the kaempferol content across the RDW samples in this study, except for the treatments IA and GA, where higher kaempferol content was detected (16.23 ± 1.136 mg/L RDW and 12.16 ± 0.851 mg/L RDW, respectively).

Kaempferol-3-*O*-glucoside, also known as astragalin, is a glycosidic form of kaempferol where a glucose molecule is attached at the 3-hydroxyl position. Glycosylation is a common modification for flavonoids in plants, primarily affecting their solubility, stability, bioavailability, and cellular localization. While the aglycone (kaempferol in this case) is often the most studied for its direct biological activity, the glycosides are the forms most commonly found and transported within the plant [57]. This study coherently identified significantly higher content of kaempferol-3-*O*-glucoside compared to its aglycone, ranging from 51.26 ± 2.050 mg/L RDW in PA to 660.0 ± 26.40 mg/L RDW in IA. Since glycosylation makes flavonoids more water-soluble, this increased solubility is crucial for their transport and accumulation within different plant tissues (e.g., vacuoles, cell walls) and for their translocation throughout the plant [58]. While the aglycone (kaempferol) might be the active form at a cellular target, the glycoside (kaempferol-3-*O*-glucoside) often serves as the transport and storage form [59]. The plant might de-glycosylate it to release the active aglycone when and where needed [60]. Therefore, applying kaempferol-3-*O*-glucoside could be a way to deliver kaempferol in a more soluble and potentially more stable form to plant tissues.

Quercetin is a flavonoid that has been extensively investigated for its plant growth-promoting effects, primarily due to its potent antioxidant properties, its role in stress tolerance, and its influence on plant physiological processes. It is a highly effective scavenger of reactive oxygen species (ROS), thus helping plants to cope with abiotic stresses (like salinity, drought, heavy metals exposure, UV radiation, heat, cold, and even flooding), leading to an overproduction of ROS and causing oxidative damage to plant cells. Therefore, quercetin contributes to the reduction in oxidative stress by directly scavenging free radicals and enhancing the activity of intrinsic plant antioxidant enzymes (e.g., superoxide dismutase, catalase, ascorbate peroxidase, glutathione reductase) [61], thus protecting cellular components by reducing the damage to cell membranes (reduced lipid peroxidation), proteins (reduced protein carbonylation) and DNA, simultaneously helping to maintain photosynthetic efficiency under stress by protecting photosynthetic pigments (chlorophylls) and improving gas exchange parameters (stomatal conductance, CO_2_ assimilation) [62]. As a direct consequence of mitigating stress, plants treated with quercetin often show improved growth parameters (shoot and root length, biomass, fresh/dry weight) even under adverse conditions. This has been demonstrated in various crops, including tomato (under salt stress) [63], *Virginia mallow* (under salt stress) [64], and fenugreek (under chromium toxicity) [65]. Quercetin has also been shown to facilitate seed germination due to its antioxidant properties that likely help to protect the embryo during the early stages of imbibition and radicle emergence [66]. It is shown to be beneficial in later plant growth phases too, promoting pollen germination and pollen tube growth, which are essential for successful fertilization and fruit set [67]. Flavonoids, including quercetin, are known to act as signaling molecules in plants. While not a classical plant hormone itself, quercetin can influence hormonal pathways; for example, by reducing stress-induced hormonal imbalances or interacting with auxin transport [68]. Quercetin also contributes to plant defense against pathogens (bacteria, fungi) and pests. It can directly inhibit microbial growth or trigger plant immune responses (e.g., by increasing salicylic acid biosynthesis), indirectly promoting healthier plant growth by reducing disease pressure [69]. Quercetin has been previously documented for its positive effects on tomato plants under abiotic stress. Exogenous quercetin application (foliar or seed priming) of concentration 15–25 µM (corresponding to 4.5–7.6 mg/L) has been shown to improve tomato plant growth (shoot/root biomass, length), increase chlorophyll content, enhance photosynthetic efficiency, and boost antioxidant enzyme activities under saline conditions [63]. The similar content of quercetin was identified in RDW samples in this study, except for the PA treatment (25.73 ± 7.718 mg/L RDW).

As for the kaempferol and kaempferol-3-*O*-glucoside, this study has identified significantly higher content of glycosidic forms of quercetin in RDW samples. Quercetin-3-*O*-glucoside, or isoquercetin, is a prominent glycosidic form of quercetin, where a glucose molecule is attached at the 3-hydroxyl position. Similarly to kaempferol-3-*O*-glucoside, its presence is widespread in plants, and its biological activities are expected to be closely related to its aglycone, quercetin. Furthermore, its antifungal activity was previously investigated, showing the ability to disturb the membrane structure of fungal cells [70]. Quercetin-3-*O*-galactoside, often referred to as hyperin or hyperoside, is another important glycosidic form of quercetin, having a galactose sugar attached at the 3-hydroxyl position. Its biological activities and PGP effects largely mirror those of its aglycone (quercetin) and its isomer (isoquercetin), primarily centered around its powerful antioxidant capacity and its role in plant stress responses. Furthermore, its roles in promoting the growth of pollen tube, as well as in modulating anthocyanin accumulation in plants, were previously confirmed [71,72,73]. Quercitrin (quercetin-3-*O*-rhamnoside) is yet another important glycosidic form of quercetin, with a rhamnose sugar attached at the 3-hydroxyl position, which has been underinvestigated in scientific literature in the context of plant growth modulation.

Rutin (quercetin-3-*O*-rutinoside) is one of the most abundant and well-studied flavonoid glycosides in nature. It consists of quercetin (the aglycone) attached to a disaccharide rutinose (glucose + rhamnose). Given its widespread occurrence in many fruits, vegetables, and medicinal plants, it has been extensively investigated for various biological activities, including PGP effects. Exogenous application of rutin has been shown to boost the plant’s own enzymatic and non-enzymatic antioxidant defense systems, especially under osmotic [74] and salinity stress [75]. It interacts with ion homeostasis regulation under salinity stress, affecting retention and exclusion of K^+^/Na^+^ ions in the specific plant tissues [76]. On the other hand, it contributes to improved photosynthetic activity and chlorophyll metabolism under osmotic stress [77]. Indirect and direct plant growth-promotion mechanisms of rutin were observed via improved germination in legume *Medicago truncatula* [66], root development in tobacco [78], as well as via development of bast fiber in *Cannabis sativa* [79]. Rutin can ‘prime’ plants, meaning it pre-activates defense mechanisms or enhances their readiness to respond to subsequent stress, leading to more robust plant resistance, especially towards bacterial [80] or insect pathogens [81]. Rutin has been evaluated as a plant biostimulant in a dosage range often set to 1–5 µM (0.6–3 mg/L), while a slightly higher rutin content range was found in various RDW samples in this study, except for treatment MIF, where the rutin content of 23.27 ± 0.698 mg/L RDW was measured.

### 4.4. Commercial and Application Outlook on RDW as a Possible Biostimulant/Seed Treatment Agent

Rose cultivation is a profitable enterprise, with a global market size of USD 560 million in 2024 [82]. However, it is also a cost-intensive activity, with major expenses allocated to human labor, land use, and, critically, fertilizers and insecticides [83]. A key economic opportunity lies in the fact that RDW represents a by-product of rose processing into essential oils, with significant environmental concerns related to its disposal. Its valorization would create a new, high-margin revenue stream, improving the overall profitability and sustainability of rose farming by turning a waste product into a valuable agricultural input, thus contributing to the overall extent of circularity of rose production. By reducing the need for traditional chemical inputs, RDW-based biostimulant/seed treatment agent could lower crop cultivation costs, increase the benefit–cost ratio, and align agricultural practices with the global movement toward more environmentally friendly methods.

Depending on the rose petal preparation and extraction method, concentration of bioactive compounds in rose extracts/RDW shows significant amplitude of variation [84]. This variability is a crucial consideration for developing a consistent biostimulant product. Research has shown significant differences in phytochemical content of rose extracts and RDW between different cultivars [5,30] and cultivation conditions [85], as similarly proven in this study, being dependent also on the rose growth phase [86]. This demonstrates that the selection of raw material is a primary factor in determining the final biostimulant product’s composition. The method of extraction also plays a critical role, especially when it comes to rose tissues’ upstream preparation steps. Additional variability in the final RDW chemical composition could be introduced by various methods of mechanical processing of rose tissues, significantly impacting the efficiency of bioactive compound extraction [84]. This variability indicates that a future commercial product would require standardized processing and careful cultivar and raw material selection to ensure consistent biostimulant content and efficacy. The RDW is not a uniform substance—its biostimulant effect is intrinsically linked to the specific source material and the precise parameters of its preparation. The bioactive compounds content in RDW samples recorded in this study is very much aligned with the previously reported dosage for similar compounds investigated for their PGP effects, confirming the suitability of the RDW to be further explored as a potential plant biostimulant, with a necessity to develop an appropriate dosage protocol.

However, this relatively simple RDW preparation procedure, albeit with high energy demands and synergism with the fact that it represents the side stream with currently low or no commercial value, significantly outperforms technical and economic feasibility compared to PGP-agents produced from microbial sources, as well as phytochemicals obtained using various green technologies. The necessity to use diluted RDW to avoid plant growth inhibition, as proven in this study, opens up the possibility to further lower optimal application concentration, achieving similar plant growth-promotion effects in further studies, thus strengthening the cost–benefit ratio for farmers as the final biostimulant/seed treatment agent users.

## 5. Conclusions and Future Research Recommendations

The results presented in this study have confirmed suitability of certain RDW types (namely arising from the following genotypes of *Rosa × hybrida*: PA—‘Pure Aroma’, MA—‘Magic Aroma’ and NA—‘Natural Aroma’) to be used as tomato biostimulants in concentration of 10% (*v*/*v*). On the other hand, the seed treatments based on increased RDW concentration (25% *v*/*v*) and undiluted RDW have resulted in tomato germination and growth inhibition. Therefore, additional experimental activities should investigate the broader range of lower concentrations of the presented prominent RDWs to establish their optimal concentrations for scale-up testing.

The future research should focus on deciphering and experimental investigation and validation of the underlying mechanisms responsible for plant growth modulating activity of RDW, some of which were proposed and discussed in this study. The influence of RDW on plant immunity should also be investigated by screening the antioxidant-linked enzyme activity in plants in vitro and at molecular level (including catalase, peroxidase, superoxide dismutase activity, etc.), simultaneously with monitoring the influence on the plant hormonal status (e.g., via auxing-responsive gene markers). The proposed application method in this study is based on seed treatment; however, additional application methods such as foliar spraying, soil spraying or soil drenching should also be investigated in the future in vitro greenhouse and field tests, followed by a precise dosage recommendations and wider range of crops included in the biostimulant screening. The major challenges in future research activities are foreseen due to variability in composition of RDW arising from varying rose growth conditions and, consequently, variability in rose chemical composition, affecting the final chemical profile of RDW. There is also a necessity to harmonize/standardize the rose petals’ hydrodistillation conditions in order to minimize variability of RDW composition arising from the experimental setup. Based on the available literature data, significant antimicrobial potential could be expected when it comes to RDW; hence, the biocontrol potential of RDW should be considered to possibly develop novel biopesticides, with targeted action not only against bacterial and fungal plant pathogens but also with possible acaricidal, insecticidal and herbicidal activity. The results of this study therefore present a solid basis for further development of novel types of plant biostimulants based on rose processing by-products, introducing a novel variable in the interplay of bio-based and chemical solutions for plant growth promotion.

## Figures and Tables

**Table 1:** pH value and electrical conductivity (EC) of rose distillation wastewater samples (100%) and their corresponding dilutions (25% and 10%, *v*/*v*).

	PA	MA	IA	NA	AA	UA	GA	MIF
pH value								
100%	4.11	4.31	4.43	4.21	4.67	4.30	4.92	4.11
25%	4.21	4.38	4.46	4.23	4.79	4.35	4.95	4.18
10%	4.35	4.40	4.54	4.32	4.89	4.39	4.98	4.22
EC (µS/cm)								
100%	612.0	590.0	725.0	555.0	661.0	565.0	669.0	606.0
25%	187.0	182.0	209.0	173.9	199.0	170.2	212.0	183.4
10%	94.8	84.5	97.3	78.9	88.5	78.3	90.2	83.4

PA—‘Pure Aroma’, MA—‘Magic Aroma’, IA—‘Intense Aroma’, NA—‘Natural Aroma’, AA—‘Adore Aroma’, UA—‘Unique Aroma’, GA—‘Gentle Aroma’, MIF—‘Mina Frayla’.

**Table 2:** Total phenolic, flavonoid and monomeric anthocyanin contents of rose distillation wastewater samples.

	PA	MA	IA	NA	AA	UA	GA	MIF
TPC								
mg GAE/g de	193.3 ± 6.309 a	150.1 ± 6.762 b	80.19 ± 2.702 d	108.6 ± 1.209 c	150.9 ± 9.834 b	78.97 ± 3.752 d	119.3 ± 1.949 b	143.3 ± 2.876 b
mg GAE/L RDW	3035 ± 99.04 a	2101 ± 94.66 c	1195 ± 40.26 e	1477 ± 16.45 d	2233 ± 145.5 bc	1208 ± 57.41 e	1503 ± 24.56 d	2336 ± 46.89 b
TFC								
mg QE/g de	24.64 ± 1.170 b	16.22 ± 0.823 c	12.54 ± 0.661 d	10.73 ± 0.180 d	26.64 ± 1.129 a	11.93 ± 0.589 d	12.31 ± 0.062 d	16.61 ± 0.225 c
mg QE/L RDW	386.8 ± 18.37 a	227.1 ± 11.52 c	186.9 ± 9.850 d	145.9 ± 2.447 e	394.3 ± 16.71 a	182.6 ± 9.143 d	155.1 ± 0.781 de	270.8 ± 3.670 b
TAC								
mg CE/g de	0.684 ± 0.043 a	<0.200	<0.200	<0.200	<0.400	<0.200	<0.200	0.230 ± 0.004 b
mg CE/L RDW	10.74 ± 0.670 a	<2.800	<2.980	<2.720	<5.920	<3.060	<2.722	3.753 ± 0.068 b

Letters a–e denote significant difference between decocts according to one-factor ANOVA followed by Tukey-HSD test (*p* ≤ 0.05). TPC—total phenolic content, GAE—gallic acid equivalents, de—dry extract, RDW—rose distillation wastewater, TFC—total flavonoid content, QE—quercetin equivalents, TAC—total monomeric anthocyanin content, CE—cyanidin 3–O–glucoside, PA—‘Pure Aroma’, MA—‘Magic Aroma’, IA—‘Intense Aroma’, NA—‘Natural Aroma’, AA—‘Adore Aroma’, UA—‘Unique Aroma’, GA—‘Gentle Aroma’, MIF—‘Mina Frayla’.

**Table 3:** Content of selected compounds in rose distillation wastewater samples determined by LC-MS/MS.

	PA	MA	IA	NA	AA	UA	GA	MIF
*p*-OH Benzoic acid								
[μg/g de]	7.796 ± 0.468 e	58.63 ± 3.518 b **	66.82 ± 4.009 b	81.58 ± 4.895 a	30.04 ± 1.802 d	24.96 ± 1.498 d	50.30 ± 3.018 c	6.823 ± 0.409 e
[mg/L RDW]	0.122 ± 0.007 e	0.821 ± 0.049 b	0.996 ± 0.060 a	1.109 ± 0.067 a	0.455 ± 0.027 d	0.382 ± 0.023 d	0.634 ± 0.038 c	0.111 ± 0.007 e
Protocatechuic acid								
[μg/g de]	3379 ± 270.3 a	80.88 ± 6.471 c	20.92 ± 1.674 c	18.65 ± 1.492 c	2645 ± 211.4 b	39.64 ± 3.171 c	15.91 ± 1.273 c	144.7 ± 11.58 c
[mg/L RDW]	53.05 ± 4.244 a	1.132 ± 0.091 c	0.312 ± 0.025 c	0.254 ± 0.020 c	39.11 ± 3.129 b	0.607 ± 0.049 c	0.201 ± 0.016 c	2.359 ± 0.189 c
*p*-Coumaric acid								
[μg/g de]	7.859 ± 0.707 c	26.96 ± 2.426 a	31.63 ± 2.847 a	19.59 ± 1.763 b	31.75 ± 2.857 a	10.05 ± 0.904 c	20.58 ± 1.852 b	6.637 ± 0.597 c
[mg/L RDW]	0.123 ± 0.011 d	0.377 ± 0.034 b	0.471 ± 0.042 a	0.266 ± 0.024 c	0.470 ± 0.042 a	0.154 ± 0.014 d	0.259 ± 0.023 c	0.108 ± 0.010 d
Vanillic acid								
[μg/g de]	<4.900 ***	<4.900	8.724 ± 2.617 a	Nd ****	<4.900	8.527 ± 2.558 a	<4.900	10.89 ± 3.268 a
[mg/L RDW]	<0.077	<0.069	0.130 ± 0.039 a	nd	<0.073	0.130 ± 0.039 a	<0.062	0.178 ± 0.053 a
Gallic acid								
[μg/g de]	76,479 ± 6883 d	151,387 ± 13,625 ab	43,632 ± 3927 e	93,771 ± 8439 cd	149,985 ± 13,499 ab	114,868 ± 10,338 c	121,277 ± 10,915 bc	157,329 ± 14,160 a
[mg/L RDW]	1201 ± 108.1 d	2119 ± 190.7 ab	650.1 ± 58.51 e	1275 ± 114.8 d	2220 ± 199.8 a	1757 ± 158.2 bc	1528 ± 255.2 cd	2564 ± 230.8 a
Caffeic acid								
[μg/g de]	<2.450	<2.450	3.247 ± 0.227	<2.450	<2.450	<2.450	<2.450	<2.450
[mg/L RDW]	<0.038	<0.034	0.048 ± 0.003	<0.033	<0.036	<0.037	<0.031	<0.040
Quinic acid								
[μg/g de]	20,701 ± 2070 e	17,250 ± 1725 e	25,231 ± 2523 de	27,389 ± 2739 cde	33,213 ± 3321 cd	37,096 ± 3710 c	81,751 ± 8175 a	50,040 ± 5004 b
[mg/L RDW]	325.0 ± 32.50 e	241.5 ± 24.15 e	375.9 ± 37.59 de	372.5 ± 37.25 de	491.6 ± 49.16 cd	567.6 ± 56.76 d	1030 ± 103.0 a	815.6 ± 81.56 b
Ferulic acid								
[μg/g de]	<2.450	<2.450	4.124 ± 0.412 b	<2.450	3.712 ± 0.371 b	3.666 ± 0.367 b	6.050 ± 0.605 a	3.437 ± 0.344 b
[mg/L RDW]	<0.038	<0.034	0.061 ± 0.006 ab	<0.033	0.055 ± 0.005 b	0.056 ± 0.006 b	0.076 ± 0.008 a	0.056 ± 0.006 b
Sinapic acid								
[μg/g de]	<9.750	<9.750	27.25 ± 2.725 a	nd	<9.750	<9.750	28.12 ± 2.812 a	nd
[mg/L RDW]	<0.153	<0.137	0.406 ± 0.041 a	nd	<0.144	<0.149	0.354 ± 0.035 b	nd
Baicalein								
[μg/g de]	<39.05	<39.05	<39.05	nd	<39.05	<39.05	53.13 ± 15.94	<39.05
[mg/L RDW]	<0.613	<0.547	<0.582	nd	<0.578	<0.597	0.669 ± 0.201	<0.637
Naringenin								
[μg/g de]	1.477 ± 0.103 d	1.355 ± 0.095 d	3.019 ± 0.211 c	3.990 ± 0.279 b	6.648 ± 0.465 a	3.613 ± 0.253 bc	4.123 ± 0.289 b	1.185 ± 0.083 d
[mg/L RDW]	0.023 ± 0.002 d	0.019 ± 0.001 d	0.045 ± 0.003 c	0.054 ± 0.004 bc	0.098 ± 0.007 a	0.055 ± 0.004 b	0.052 ± 0.004 bc	0.019 ± 0.001 d
Kaempferol								
[μg/g de]	115.8 ± 8.106 d	57.35 ± 4.015 d	1089 ± 76.23 a	350.2 ± 24.52 c	287.2 ± 20.11 c	162.1 ± 11.35 d	964.9 ± 67.54 b	155.1 ± 10.86 d
[mg/L RDW]	1.818 ± 0.127 de	0.803 ± 0.056 e	16.23 ± 1.136 a	4.763 ± 0.333 c	4.251 ± 0.298 c	2.480 ± 0.174 d	12.16 ± 0.851 b	2.528 ± 0.177 d
Catechin								
[μg/g de]	nd	nd	203.7 ± 20.37 a	47.22 ± 4.722 c	114.7 ± 11.47 b	194.5 ± 19.45 a	57.07 ± 5.707 c	176.2 ± 17.62 a
[mg/L RDW]	nd	nd	3.035 ± 0.304 a	0.642 ± 0.064 c	1.698 ± 0.170 b	2.975 ± 0.298 a	0.719 ± 0.072 c	2.873 ± 0.287 a
Chrysoeriol								
[μg/g de]	<0.300	<0.300	<0.300	<0.300	<0.300	<0.300	<0.300	0.826 ± 0.025
[mg/L RDW]	<0.005	<0.004	<0.004	<0.004	<0.004	<0.005	<0.004	0.014 ± 0.0004
Quercetin								
[μg/g de]	1639 ± 491.6 a	239.8 ± 71.94 b	112.1 ± 33.64 b	61.37 ± 18.41 b	358.9 ± 107.7 b	92.97 ± 27.89 b	63.27 ± 18.98 b	429.9 ± 129.0 b
[mg/L RDW]	25.73 ± 7.718 a	3.357 ± 1.007 b	1.671 ± 0.501 b	0.835 ± 0.250 b	5.312 ± 1.594 b	1.423 ± 0.427 b	0.797 ± 0.239 b	7.007 ± 2.102 b
Chlorogenic acid								
[μg/g de]	<4.900	<4.900	10.11 ± 0.506 a	<4.900	<4.900	<4.900	<4.900	9.105 ± 0.455 a
[mg/L RDW]	<0. 77	<0.069	0.151 ± 0.008 a	<0.067	<0.073	<0.075	<0.062	0.148 ± 0.007 a
Kaempherol 3–*O*–Glc								
[μg/g de]	3265 ± 130.6 f	4500 ± 180.0 f	44,295 ± 1772 a	23,813 ± 952.5 c	19,884 ± 795.4 d	27,167 ± 1087 b	23,631 ± 945.3 c	9298 ± 371.9 e
[mg/L RDW]	51.26 ± 2.050 e	63.00 ± 2.520 e	660.0 ± 26.40 a	323.9 ± 12.95 c	294.3 ± 11.77 c	415.7 ± 16.63 b	297.8 ± 11.91 c	151.6 ± 6.062 d
Quercitrin								
[μg/g de]	14,095 ± 845.7 b	11,304 ± 678.2 c	1208 ± 72.46 fg	2411 ± 144.7 ef	16,908 ± 1014 a	2802 ± 168.1 e	159.9 ± 9.591 g	8535 ± 512.1 d
[mg/L RDW]	221.3 ± 13.28 b	158.3 ± 9.495 c	17.99 ± 1.080 ef	32.79 ± 1.968 de	250.2 ± 15.01 a	42.87 ± 2.572 d	2.014 ± 0.121 f	139.1 ± 8.347 c
Hyperoside + Isoquercetin								
[μg/g de]	9202 ± 552.1 b	7040 ± 422.4 c	4785 ± 287.1 d	1865 ± 111.9 e	16,655 ± 999.3 a	4076 ± 244.6 d	587.7 ± 35.26 e	9331 ± 559.8 b
[mg/L RDW]	144.5 ± 8.668 b	98.57 ± 5.914 c	71.30 ± 4.278 d	25.36 ± 1.522 e	246.5 ± 14.79 a	62.37 ± 3.742 d	7.405 ± 0.444 e	152.1 ± 9.125 b
Amenthoflavon								
[μg/g de]	1.759 ± 0.053	<1.200	<1.200	<1.200	<1.200	<1.200	<1.200	<1.200
[mg/L RDW]	0.028 ± 0.001	<0.017	<0.018	<0.016	<0.018	<0.018	<0.015	<0.020
Rutin								
[μg/g de]	461.9 ± 13.86 b	392.0 ± 11.76 cd	190.1 ± 5.704 f	43.10 ± 1.293 g	412.3 ± 12.37 bc	315.2 ± 9.457 e	352.9 ± 10.59 de	1428 ± 42.83 a
[mg/L RDW]	7.252 ± 0.218 b	5.487 ± 0.165 cd	2.833 ± 0.085 f	0.586 ± 0.018 g	6.101 ± 0.183 c	4.823 ± 0.145 de	4.447 ± 0.133 e	23.27 ± 0.698 a
Sum of quantified compounds								
[mg/g de]	129.4	192.3	120.9	149.9	240.5	186.9	229.0	236.9
[g/L RDW]	2.031	2.692	1.802	2.037	3.560	2.677	2.885	3.861

Results are given as content ± standard error of repeatability (as determined via method validation); ** Letters within same row (a–g) denote significant difference between contents in decocts according to one-factor ANOVA followed by Tukey-HSD test (*p* ≤ 0.05); *** below the limit of quantification (LOQ), **** nd—not detected. de—dry extract, RDW—rose distillation wastewater, PA—‘Pure Aroma’, MA—‘Magic Aroma’, IA—‘Intense Aroma’, NA—‘Natural Aroma’, AA—‘Adore Aroma’, UA—‘Unique Aroma’, GA—‘Gentle Aroma’, MIF—‘Mina Frayla’.

**Table 4:** In vitro antioxidants properties of rose distillation wastewater samples.

	PA	MA	IA	NA	AA	UA	GA	MIF
DPPH								
IC_50_ [μg de/mL]	9.450 ± 0.028 de	10.57 ± 0.665 c	19.38 ± 0.531 a	9.575 ± 0.120 d	7.115 ± 0.403 f	11.67 ± 0.106 b	11.64 ± 0.226 b	8.660 ± 0.099 e
FRAP								
[mg AAE/g de]	182.0 ± 7.852 a	145.2 ± 10.29 b	83.31 ± 3.820 d	101.4 ± 5.956 d	140.0 ± 6.718 bc	122.9 ± 8.604 c	127.2 ± 4.135 bc	183.2 ± 9.668 a

Letters within same row denote statistically significant difference between decocts according to one-factor ANOVA followed by Tukey-HSD test (*p* ≤ 0.05); IC_50_—the concentration of the extract that neutralizes 50% of DPPH radicals, de—dry extract, AAE—ascorbic acid equivalents, PA—‘Pure Aroma’, MA—‘Magic Aroma’, IA—‘Intense Aroma’, NA—‘Natural Aroma’, AA—‘Adore Aroma’, UA—‘Unique Aroma’, GA—‘Gentle Aroma’, MIF—‘Mina Frayla’.

## Data Availability

The original contributions presented in this study are included in the article. Further inquiries can be directed at the corresponding author.

## Supplementary Materials

The following supporting information can be downloaded at https://www.mdpi.com/article/10.3390/antiox14101252/s1, Table S1. LOD (limit of detection) and LOQ (limit of quantification) of the LC-MS/MS method for quantitative determination of selected compounds, Table S2. Standard curve equations and R2 (coefficients of determination) for all quantified compounds in rose distillation wastewater by LC-MS/MS, Table S3. Tomato germination/growth parameters after 7-day seed treatment using various samples and concentrations of rose distillation wastewater (RDW).

## Abbreviations

The following abbreviations are used in this manuscript:

CE	cyanidin 3-*O*-glucoside
de	dry extract
GAE	gallic acid equivalents
AA	‘Adore Aroma’
GA	‘Gentle Aroma’
IA	‘Intense Aroma’
MA	‘Magic Aroma’
MIF	‘Mina Frayla’
NA	‘Natural Aroma’
PA	‘Pure Aroma’
UA	‘Unique Aroma’
TPC	total phenolic content
TFC	total flavonoid content
TAC	total monomeric anthocyanin content
QE	quercetin equivalents
RDW	rose distillation wastewater
PGP	plant growth promotion
GP	germination percent
MGT	mean germination time
GRI	germination rate index
SVI-I	seedling vigor index I
SVI-II	seedling vigor index II
RL	root length
SL	shoot length
TL	total seedling length
FM	fresh mass
DM	dry mass
PCA	principal component analysis
