# Supplementary material for: Rose Oil Distillation Wastewater: By-Products of Essential Oil Extraction as Circular Biostimulants for Tomato Growth
Source: Antioxidants (Basel). 2025 Oct 18;14(10):1252. doi: 10.3390/antiox14101252 (PMC12561105; doi:10.3390/antiox14101252)
Supplement: Supplementary file 1 [file antioxidants-14-01252-s001.zip › antioxidants-3917726-supplementary.pdf]

antioxidants

MDPI

Article

# Rose Oil Distillation Wastewater: By-products of Essential Oil Extraction as Circular Biostimulants for Tomato Growth

**Nemanja Živanović [1], Ivana Danilov [2,*], Marija Lesjak [1], Tatjana Dujković [2], Nataša Simin [1], Vanja Vlajkov [2], Mirjana Ljubojević [3], and Jovana Grahovac [2]**

[1] Faculty of Sciences, University of Novi Sad, Trg Dositeja Obradovića 3, 21000 Novi Sad, Serbia; nemanja.zivanovic@dh.uns.ac.rs (N.Ž.), marija.lesjak@dh.uns.ac.rs (M.L.), natasa.simin@dh.uns.ac.rs (N.S.)

[2] Faculty of Technology Novi Sad, University of Novi Sad, Bulevar cara Lazara 1, 21000 Novi Sad, Serbia; ivana.pajcin@uns.ac.rs (I.D.), tatjana.dujkovic@uns.ac.rs (T.D.), vanja.vlajkov@uns.ac.rs (V.V.), johana@uns.ac.rs (J.G.)

[3] Faculty of Agriculture, University of Novi Sad, Trg Dositeja Obradovića 8, 21000 Novi Sad, Serbia; mirjana.ljubojevic@polj.uns.ac.rs (M.Lj.)

* Correspondence: ivana.pajcin@uns.ac.rs.

## SUPPLEMENTARY MATERIAL

**Table S1.** LOD (limit of detection) and LOQ (limit of quantification) of the LC-MS/MS method for quantitative determination of selected compounds.

| Compound | LOD [ng/mL] | LOQ [ng/mL] |
|---|---|---|
| ***p*-Hydroxybenzoic acid** | 12.21 | 48.83 |
| **Cinnamic acid** | 97.66 | 195.31 |
| **Protocatechuic acid** | 1.53 | 24.41 |
| **Gentisic acid** | 1.53 | 24.41 |
| ***p*-Coumaric acid** | 3.05 | 6.10 |
| **Umbelliferon** | 1.53 | 6.10 |
| ***o*-Coumaric acid** | 12.21 | 24.41 |
| **Vanillic acid** | 97.66 | 97.66 |
| **Gallic acid** | 48.83 | 195.31 |
| **Esculetin** | 6.10 | 48.83 |
| **Caffeic acid** | 12.21 | 48.83 |
| **Quinic acid** | 48.83 | 195.31 |
| **Scopoletin** | 3.05 | 24.41 |
| **Ferulic acid** | 24.41 | 48.83 |
| **Syringic acid** | 97.66 | 195.31 |
| **3,4-Dimethoxycinnamic acid** | 97.66 | 97.66 |
| **Sinapic acid** | 195.31 | 195.31 |
| **Daidzein** | 12.21 | 24.41 |
| **Apigenin** | 1.53 | 12.21 |
| **Genistein** | 3.05 | 12.21 |

 https://doi.org/10.3390/antiox14101252

| | | |
|---|---|---|
| **Baicalein** | 781.25 | 781.25 |
| **Naringenin** | 3.05 | 6.10 |
| **Luteolin** | 6.10 | 48.83 |
| **Kaempferol** | 48.83 | 48.83 |
| **Catechin** | 97.66 | 195.31 |
| **Epicatechin** | 97.66 | 97.66 |
| **Chrysoeryol** | 1.53 | 12.21 |
| **Quercetin** | 97.66 | 390.63 |
| **Isorhamnetin** | 12.21 | 48.83 |
| **Myricetin** | 781.25 | 1562.5 |
| **Chlorogenic acid** | 3.05 | 97.66 |
| **Matairesinol** | 48.83 | 97.66 |
| **Secoisolariciresinol** | 97.66 | 97.66 |
| **Apigenin 7-O-glucoside** | 1.53 | 24.41 |
| **Vitexin** | 1.53 | 24.41 |
| **Baicalin** | 48.83 | 48.83 |
| **Kaempferol 3-O-glucoside** | 1.53 | 24.41 |
| **Luteolin 7-O-glucoside** | 1.53 | 48.83 |
| **Quercitrin** | 1.53 | 24.41 |
| **Epigallocatechin gallate** | 781.25 | 1562.5 |
| **Isoquercetin + Hyperoside** | 3.05 | 48.83 |
| **Amenthoflavone** | 1.53 | 24.41 |
| **Apiin** | 1.53 | 24.41 |
| **Rutin** | 1.53 | 48.83 |

**Table S2.** Standard curve equations and $R^2$ (coefficients of determination) for all quantified compounds in rose distillation wastewater by LC-MS/MS.

| Compound | Standard curve equation | $R^2$ |
|---|---|---|
| ***p*-Hydroxybenzoic acid** | y = 92.61458 + 2.04308*x + (-1.30393E-4)*x^2 | 0.99835 |
| **Protocatechuic acid** | y = -1420.36985 + 6.32528*x + (-2.63602E-4)*x^2 (used for samples MA, IA, NA, UA, GA, MIF) | 0.94965 |
| | y = -337.7926 + 4.68743*x (used for samples PA, AA) | 0.99163 |
| ***p*-Coumaric acid** | y = 78.05172 + 5.2805*x | 0.99766 |
| **Vanillic acid** | y = -24.20833 + 0.25337*x | 0.97757 |
| **Gallic acid** | y = 103.01049 + 1.3545*x | 0.99091 |
| **Caffeic acid** | y = -93.61692 + 8.01701*x | 0.99757 |
| **Quinic acid** | y = -6.95799 + 0.40497*x | 0.99342 |
| **Ferulic acid** | y = -19.95833 + 1.09059*x | 0.99791 |
| **Sinapic acid** | y = -57.91667 + 0.34297*x | 0.99617 |
| **Baicalein** | y = -7619.39216 + 6.90904*x + 9.74303E-4*x^2 | 0.99751 |
| **Naringenin** | y = 9.35632 + 4.11858*x | 0.9975 |
| **Kaempferol** | y = -1417.16667 + 20.38257*x (used for samples PA, MA, NA, AA, UA, MIF) | 0.99639 |
| | y = 7262.30348 + 16.34719*x (used for samples IA and GA) | 0.98041 |
| **Catechin** | y = -0.48281 + 0.14524*x | 0.98159 |
| **Chrysoeryol** | y = -26.70647 + 12.76974*x | 0.99947 |

| | | |
|---|---|---|
| **Quercetin** | y = -1170.19403 + 3.94745*x (used for samples MA, IA, NA, AA, UA, GA, MIF) | 0.99743 |
| | y = -641.375 + 4.39393*x (used for sample PA) | 0.99402 |
| **Chlorogenic acid** | y = -272.875 + 3.46983*x | 0.96434 |
| **Kaempferol 3-O-glucoside** | y = 63.86885 + 6.29464*x + (-4.70877E-5)*x^2 | 0.99963 |
| **Quercitrin** | y = -3445.31366 + 12.55282*x + (-1.03816E-4)*x^2 (used for sample GA) | 0.99816 |
| | y = 28.58122 + 14.59956*x + (-1.20311E-4)*x^2 (used for samples PA, MA, IA, NA, AA, UA, MIF) | 0.99983 |
| **Isoquercetin + Hyperoside** | y = -4615.39205 + 9.9008*x + (-8.33842E-5)*x^2 (used for sample GA) | 0.99842 |
| | y = -41.02822 + 13.45432*x + (-2.24832E-4)*x^2 (used for samples PA, MA, IA, NA, AA, UA, MIF) | 0.99988 |
| **Amenthoflavone** | y = -442.64705 + 41.18906*x + (-0.04894)*x^2 | 0.98951 |
| **Rutin** | y = -369.89277 + 6.04156*x + (-1.00028E-5)*x^2 (used for samples PA, MA, IA, NA, AA, UA, GA) | 0.99953 |
| | y = -94.82708 + 8.9445*x + (-3.04465E-4)*x^2 (used for sample MIF) | 0.99974 |

The results of the tomato growth promotion parameters measured after the treatments using 25% and 100% (v/v) RDW samples are presented in Table S3. As presented in the table, the increase of the RDW concentration in comparison to the results presented in Figure 1 and Figure 2 has resulted in the decrease of almost all recorded plant growth parameters in comparison to the RDW concentration 10% (v/v), suggesting the tomato growth inhibition by the increased concentration of the compounds present in RDW samples.

**Table S3.** Tomato germination/growth parameters after 7-day seed treatment using various samples and concentrations of rose distillation wastewater (RDW).

| | PA | MA | IA | NA | AA | UA | GA | MIF |
|---|---|---|---|---|---|---|---|---|
| **RDW concentration 25% (v/v)** | | | | | | | | |
| **RL (mm)** | 36.00 ± 20.04 | 52.80 ± 40.31 | 15.20 ± 9.52 | 29.20 ± 11.82 | 51.60 ± 30.84 | 12.20 ± 7.09 | 32.20 ± 11.28 | 19.60 ± 18.58 |
| **SL (mm)** | 32.80 ± 12.56 | 29.00 ± 21.92 | 20.20 ± 12.01 | 32.00 ± 14.63 | 30.40 ± 18.77 | 24.00 ± 17.33 | 31.40 ± 7.50 | 17.00 ± 16.34 |
| **TL (mm)** | 68.80 ± 29.07 | 81.80 ± 61.76 | 35.40 ± 21.41 | 61.20 ± 25.96 | 82.00 ± 46.83 | 36.20 ± 24.08 | 63.60 ± 17.01 | 36.60 ± 34.85 |
| **GP (mm)** | 100.00 ± 0.00 | 80.00 ± 0.00 | 80.00 ± 0.00 | 100.00 ± 0.00 | 80.00 ± 0.00 | 100.00 ± 0.00 | 100.00 ± 0.00 | 60.00 ± 0.00 |
| **FM (g)** | 0.0286 ± 0.0000 | 0.0286 ± 0.0020 | 0.0236 ± 0.0026 | 0.0309 ± 0.0000 | 0.0346 ± 0.0037 | 0.0266 ± 0.0000 | 0.0331 ± 0.0000 | 0.0236 ± 0.0032 |

| | | | | | | | | |
|---|---|---|---|---|---|---|---|---|
| **DM (g)** | 0.0022 ± 0.0000 | 0.0017 ± 0.0002 | 0.0012 ± 0.0000 | 0.0019 ± 0.0000 | 0.0027 ± 0.0003 | 0.0018 ± 0.0000 | 0.0016 ± 0.0000 | 0.0021 ± 0.0003 |
| **MGT (day)** | 3.84 ± 0.05 | 3.76 ± 0.02 | 4.27 ± 0.03 | 4.42 ± 0.04 | 2.78 ± 0.07 | 4.20 ± 0.00 | 3.42 ± 0.04 | 3.70 ± 0.18 |
| **GRI (seeds/day)** | 1.42 ± 0.03 | 1.18 ± 0.04 | 0.96 ± 0.02 | 1.20 ± 0.00 | 1.50 ± 0.04 | 1.38 ± 0.01 | 1.63 ± 0.02 | 0.96 ± 0.02 |
| **SVI-I** | 688.00 ± 290.72 | 654.40 ± 494.11 | 283.20 ± 171.26 | 612.00 ± 259.56 | 656.00 ± 374.68 | 362.00 ± 240.77 | 636.00 ± 170.09 | 219.60 ± 209.08 |
| **SVI-II** | 218.00 ± 2.83 | 134.72 ± 17.17 | 92.48 ± 6.44 | 189.20 ± 1.79 | 216.96 ± 22.90 | 175.20 ± 1.64 | 158.60 ± 2.41 | 127.44 ± 0.84 |
| **RDW concentration 100% (v/v)** | | | | | | | | |
| **RL (mm)** | 22.20 ± 17.85 | 32.00 ± 28.26 | 26.80 ± 24.53 | 7.67 ± 7.60 | 15.80 ± 14.45 | 9.00 ± 5.70 | 5.20 ± 3.11 | 13.27 ± 11.80 |
| **SL (mm)** | 13.60 ± 8.20 | 12.00 ± 10.20 | 23.40 ± 7.80 | 7.60 ± 7.09 | 13.80 ± 2.95 | 11.20 ± 7.40 | 9.80 ± 3.56 | 15.74 ± 15.40 |
| **TL (mm)** | 35.80 ± 25.21 | 44.00 ± 38.35 | 50.20 ± 28.23 | 15.20 ± 14.24 | 29.60 ± 16.58 | 20.20 ± 11.37 | 15.00 ± 6.34 | 27.20 ± 25.41 |
| **GP (mm)** | 100.00 ± 0.00 | 80.00 ± 0.00 | 100.00 ± 0.00 | 60.00 ± 0.00 | 100.00 ± 0.00 | 80.00 ± 0.00 | 100.00 ± 0.00 | 100.00 ± 0.00 |
| **FM (g)** | 0.0168 ± 0.0000 | 0.0194 ± 0.0025 | 0.0296 ± 0.0000 | 0.0131 ± 0.0027 | 0.0194 ± 0.0003 | 0.0166 ± 0.0031 | 0.0201 ± 0.0009 | 0.0160 ± 0.0003 |
| **DM (g)** | 0.0022 ± 0.0000 | 0.0026 ± 0.0003 | 0.0024 ± 0.0000 | 0.0021 ± 0.0006 | 0.0027 ± 0.0000 | 0.0020 ± 0.0003 | 0.0024 ± 0.0002 | 0.0028 ± 0.0001 |
| **MGT (day)** | 4.18 ± 0.04 | 4.02 ± 0.04 | 3.40 ± 0.00 | 4.04 ± 0.05 | 4.44 ± 0.05 | 4.00 ± 0.00 | 5.46 ± 0.05 | 5.42 ± 0.04 |
| **GRI (seeds/day)** | 1.42 ± 0.04 | 1.12 ± 0.03 | 1.61 ± 0.01 | 0.78 ± 0.01 | 1.20 ± 0.00 | 1.01 ± 0.01 | 0.95 ± 0.04 | 1.02 ± 0.04 |
| **SVI-I** | 357.70 ± 252.44 | 351.60 ± 307.09 | 501.24 ± 282.76 | 90.70 ± 85.00 | 295.60 ± 166.06 | 161.12 ± 90.74 | 149.76 ± 60.57 | 271.82 ± 254.27 |
| **SVI-II** | 224.16 ± 4.44 | 203.94 ± 24.66 | 237.13 ± 2.79 | 127.77 ± 34.50 | 265.87 ± 3.92 | 156.70 ± 20.54 | 241.50 ± 17.64 | 277.46 ± 11.50 |

PA – 'Pure Aroma', MA – 'Magic Aroma', IA – 'Intense Aroma', NA – 'Natural Aroma', AA – 'Adore Aroma', UA – 'Unique Aroma', GA – 'Gentle Aroma', MIF – 'Mina Frayla'. RL – root length, SL – shoot length, TL – total seedling length, FM – fresh mass, DM – dry mass, GP - germination percent, MGT - mean germination time, GRI – germination rate index, SVI-I and SVI-II - seedling vigor indices I and II.
